# Genetic exchange in *Trypanosoma brucei*: Evidence for mating prior to metacyclic stage development

**DOI:** 10.1016/j.molbiopara.2006.10.009

**Published:** 2007-01

**Authors:** Andy Tait, Annette MacLeod, Alison Tweedie, Dan Masiga, C. Michael R. Turner

**Affiliations:** a Wellcome Centre for Molecular Parasitology, University of Glasgow, Glasgow Biomedical Research Centre, 120, University Place, Glasgow G12 8TA, UK; b Division of Infection and Immunity, IBLS, University of Glasgow, Glasgow Biomedical Research Centre, 120, University Place, Glasgow G12 8TA, UK

**Keywords:** *Trypanosoma brucei*, Genetic exchange, Life cycle stage, AFLP, amplified fragment length polymorphism, TREU, trypanosomiasis research, Edinburgh University, STIB, Swiss Tropical Institute, Basel, PCR, polymerase chain reaction

It is well established that genetic exchange occurs between *Trypanosoma brucei* parasites when two stocks are used to infect tsetse flies under laboratory conditions and a number of such crosses have been undertaken [1,2]. Both cross and self-fertilisation can take place [3,4], with the products of mating being the equivalent of F1 progeny in a Mendelian system [2,3]. Recently, analysis of a large collection of independent progeny using a series of polymorphic micro and minisatellite markers, has formally demonstrated that the allelic segregation at loci on each of the 11-megabase chromosomes conforms to ratios predicted for a classical diploid genetic system involving meiosis as well as independent assortment of markers on different chromosomes [5]. Further extensive analysis of these F1 progeny, using a large panel of micro and minisatellite markers, has led to the construction of a genetic map of one parasite stock [6].

One of the remaining unknowns about this system of genetic exchange is the life cycle stage at which mating takes place. Mating clearly takes place in the tsetse fly [1,2] and in principal could take place at any point during cyclical development. In the tsetse, procyclic form trypanosomes migrate anteriorly from the mid-gut into the foregut and proboscis of a tsetse fly and thence to the salivary glands, from where free-swimming mature metacyclic forms are expelled with saliva during tsetse feeding. The development from procyclic to metacyclic is via a succession of morphologically distinct stages, the most prominent of which are the mesocyclic and epimastogote stages in the proventiculus and salivary glands, respectively [7,8]. Gibson and Bailey [9], using parental stocks tagged with selectable markers, provided evidence that mating does not occur between procyclic stage trypanosomes in the mid-gut of the tsetse fly, in contrast to a previous report [10]. Using an innovative approach involving GFP tagging, the Gibson group showed that the products of mating only occur in the salivary glands although the precise stage in trypanosome development was not specified [11]. The genotypes of single metacyclic trypanosomes (amplified vegetatively in mice) obtained from crosses, clearly show that they are the products of mating [1,3]. Taken together, the available evidence is consistent with mating taking place at one of three possible life cycle stages in the tsetse fly: (1) between metacyclic forms, (2) between epimastigotes, or (3) at a pre-epimastigote stage post the procyclic stage in the mid-gut.

To test the possibility that mating occurs between metacyclic stage trypanosomes, we genotyped a series of progeny clones derived from single metacyclic stage trypanosomes taken from tsetse flies infected with mating mixtures of two pairs of parental stocks (TREU 927 × STIB 247 or STIB 386 × STIB 247). Single metacyclic trypanosomes were isolated from either dissected salivary glands or guinea pig serum into which infected tsetse had been allowed to probe, each expanded by injection into a single mouse and the resulting infected blood cryopreserved. The metacyclic stage trypanosomes were isolated from two flies (F532 and F974) in the TREU 927/STIB 247 cross and two flies in the STIB 386/STIB 247 cross (F9 and F492) These progeny have been described previously and been shown to be hybrid for a series of RFLP and iso-enzyme markers [3,12], indicating that they are the products of genetic exchange between the parental stocks. The cryopreserved metacyclic clones were expanded in immuno-suppressed mice and DNA prepared from purified trypanosomes as previously described [3]. A total of 11 progeny clones from the first cross and 9 from the second were generated (listed in Table 1). Each DNA preparation of the metacyclic derived progeny clones from the cross STIB 247 × TREU 927 was genotyped with three polymorphic minisatellite markers (TB1/1, TB3/13 and TB10/14) located on chromosomes 1, 3 and 10, respectively [6] and 10 microsatellite markers (listed in Table 2) located on chromosomes 1, 2, 3, 4 and 5 [6] using the PCR conditions described previously [5]. Similarly, the preparations from the metacyclic derived progeny of the cross STIB 247 × STIB 386 were genotyped with the same three minisatellite markers and nine microsatellite markers located on chromosomes 1, 2, 3, 4, 5 and 10. The markers on different chromosomes will segregate independently [5] while those on the same chromosomes have been chosen on the basis of being separated by genetic distances (Table 2) to give a high probability of cross-over between them. The genetic distances correspond to physical distances of 185–525 kb in the sequence of TREU 927 but the physical distances have not been determined for STIB 386. The genotyping allowed the construction of a multilocus genotype (MLG 1–17) for each progeny clone and comparative analysis showed that the multilocus genotypes of the clones from the 247 × 927 cross were unique, except for clones F532/72 mcl 1 and 4 (MLG 2), while the same analysis with the progeny from the 247 × 386 cross identified two pairs of clones (F9/45 mcl 7 and F9/45 mcl 11–MLG 13; F9/45 mcl 9 and F9/45 mcl 10–MLG 14) that were identical to each other for all micro- and minisatellite markers (Table 1), although the remaining clones were unique.

To test the identity of these pairs of clones further, genome wide markers were used. Previous work, using the technique of amplified fragment length polymorphism (AFLP [13,14]), has been undertaken to construct linkage maps of TREU 927 and STIB 386 using some of the metacyclic derived progeny and provides genome wide analyses of polymorphic segregating markers [2]. DNA was prepared for AFLP analysis from the parental and progeny clones after growth in immuno-suppressed mice (independent preparations from those used for the microsatellite analysis). A section of one gel is shown in Fig. 1 to illustrate the differences observed between the parental stocks and the inheritance of these differences in the resulting progeny. The parental clones (247 and 386) show a series of identical bands as well as differences in which a band is present in one parent but not the other and some of these bands segregate in the F1 progeny clones as a presence/absence (Fig. 1 arrows). This can be interpreted as the homozygous absence of a polymorphic site in one parent (therefore no amplification product) with the second parent being heterozygous for that polymorphism. These polymorphisms arise from the presence/absence of a restriction site, insertion/deletion or single base change in the sequence matching the 3′ extension of one primer. This polymorphism will then segregate into the progeny, which will inherit one ‘absent’ allele from one parent and either the amplified band or ‘absent’ allele from the second parent [2,13]. Applying this technique to the detection of differences between the three parental clones (247, 386 and 927), using the restriction enzyme pairs *Eco*R1/*Mse*1 and *Hin*dIII/*Taq*1, and screening the F1 progeny clones to detect markers that segregate, 157 segregating polymorphic fragments were detected in the 247 × 386 cross and 185 in the 247 × 927 cross. Fig. 1 illustrates the different patterns of segregation of the parental bands in progeny that differ in genotype (lanes 3–7), as well as the identity of clones F9/45 mcl 9 and 10 (lanes 7 and 8). These data allow a detailed genetic fingerprint of each progeny clone to be generated and a comparison between the clones in terms of their similarity. The AFLP data confirmed the findings with the min- and microsatellite markers. In the cross 927 × 247, seven of the metacyclic derived clones were confirmed to be of distinct, independent genotypes and two clones were identical to each other (AFLP genotype A, Table 1). These data indicate that most of the metacyclic stage clones are genotypically distinct and that the metacyclic stage is of hybrid genotype and therefore a product of mating (Table 1). AFLP analysis of six of the metacyclic derived clones from the 247 × 386 cross, showed that five were of unique genotype and two were identical for all AFLP markers (AFLP genotype J, Table 1). When these data are combined with the multilocus genotypes generated from the micro- and minisatellite data, there is clear evidence that one pair of clones from the 927 × 247 cross and two pairs from the 386 × 247 cross are identical in terms of genotype. While a proportion of these markers will be genetically linked, there is a sufficiently large number of independent markers to make the conclusion of identity a robust one.

If mating occurred between the metacyclic stage trypanosomes the probability that two F1 progeny, in the absence of division, would be identical in the sample sizes from each cross is extremely low as the products of meiosis will all be different. The evidence that metacyclics do not divide is based on extensive electron microscopy studies of infected salivary glands, where there is ample evidence for dividing epimastigotes but no evidence for the division of either the mature or nascent metacyclics that have acquired a VSG coat [15]. Additionally, immuno-electron microscopy with monoclonal antibodies specific for a particular VSG, shows that the metacyclics expressing this VSG are randomly distributed throughout the gland and occur in isolation. If the metacyclic divided you would predict clusters of such cells expressing the same VSG-this is not observed [16]. On this basis, mating cannot be occurring between the mature metacyclic stage trypanosomes and the identical clones must have arisen by vegetative division of a single original product of mating. This result suggests that mating takes place either amongst epimastigote or pre-metacyclic forms in the salivary glands or at a stage in the foregut/proboscis. However, in both crosses most metacyclic stage clones are unique therefore suggesting there is very limited vegetative growth between mating and the development of the metacyclic otherwise many identical clones would be observed. In order to explain the results from the two crosses, taken together, mating cannot take place between the metacyclic stages but is most likely to occur between the epimastigotes with limited subsequent mitotic division between mating and the development of the metacyclic stage. If mating occurred prior to the epimastigote stage between some of the morphologically distinct stages identified in the foregut and proboscis [7], the subsequent multiple rounds of division of the epimastigotes [7,8] would lead to many of the metacyclic stage trypanosomes being of identical genotype. This is clearly not the case. Our data strongly support mating and meiosis occurring between attached epimastigotes or dividing pre-metacyclic stages in the salivary glands. Two indirect arguments suggest that the epimastigote is the most likely stage at which mating takes place. Firstly, pre-metacyclics are relatively rare and this is thought to be a very transient phase in development [8,15]. Secondly, in other flagellates, the process of gamete fusion that leads to syngamy is first mediated via flagella attachment [17] and it is only in the attached epimastigote phase of the life cycle that *T. brucei* has the required flagellum–substratum and flagellum–flagellum interactions [18].

## Figures and Tables

**Fig. 1:**
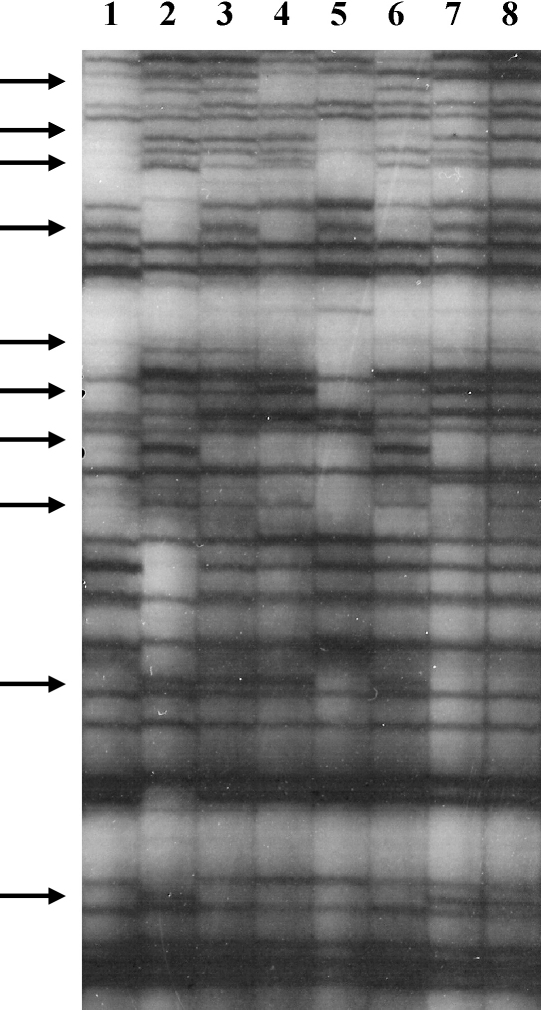
Autoradiograph of labelled AFLP gel with DNA samples digested with *Hin*dIII and *Taq*1. Each primer had a two-nucleotide extension (H-CA and T-AA) for selective amplification and fragments were separated on a 6% acrylamide gel. Tracks: 1-STIB 247; 2-STIB 386; 3-F9/45 mcl 2; 4-F9/45 mcl 11; 5-F492/50 mcl 12; 6-F492/50 mcl 13; 7-F9/45 mcl 9; 8-F9/45 mcl 10. Arrows indicate fragments that are heterozygous for polymorphisms in one of the two parental stocks and segregate in the progeny.

**Table 1:** The genotypes of the metacyclic stage F1 progeny clones from two crosses, determined by micro and minisatellite markers as well as AFLP genotyping

Cross	Clone	MLG	AFLP
247 × 927	F532/53 mcl 1	1	ND
	F532/72 mcl 1	2	A
	F532/72 mcl 2	3	B
	F532/72 mcl 3	4	C
	F532/72 mcl 4	2	A
	F532/72 mcl 5	5	D
	F532/72 mcl 6	6	E
	F532/72 mcl 7	7	F
	F532/72 mcl 9	8	G
	F532/72 mcl 8	9	ND
	F974/70 mcl 4	10	ND

247 × 386	F9/34 mcl 1	11	ND
	F9/45 mcl 2	12	H
	F9/45 mcl 7	13	ND
	F9/45 mcl 11	13	I
	F9/45 mcl 9	14	J
	F9/45 mcl 10	14	J
	F9/45 mcl 12	15	ND
	F492/50 mcl 12	16	K
	F492/50 mcl 13	17	L

Each multilocus genotype (MLG) was based on the analysis of 12–13 micro- and minisatellite markers and each MLG that was different has been given a different number. The genotypes determined by AFLP used 157 segregating markers (247 × 386) and 185 markers (247 × 927). Each AFLP that was different has been given a different letter. ND, not determined.

**Table 2:** Micro- and minisatellite markers used for genotyping

Cross	Chromosome	Marker	Genetic distance (cM)
927 × 247	1	TB 1/1	
			17.5
		TB 1/7	
			18.3
		TB 1/13	

	2	TB 2/3	
			42.7
		TB 2/10	
			38.5
		TB 2/20	

	4	TB 4/1	
			32.2
		TB 4/4	
			25.9
		TB 4/10	
			31.2
		TB 4/16	

386 × 247	1	TB 1/1	
			11.1
		TB 1/4	
			50.6
		TG 1/4	

	2	TB 2/3	
			42.7
		TB 2/10	
			38.5
		TB 2/20	

	4	TB 4/1	
			54.7
		TB 4/8	
			31.8
		TB 4/14	

The genetic distances are all based on the TREU 927 genetic map, except for marker TG1/4 which is only informative for the STIB 386/STIB 247cross. Markers TB 3/13, TB 5/4 and TB 10/14 on chromosomes 3, 5 and 10 were also used.
